# Fine-tuning the space, time, and host distribution of mycobacteria in wildlife

**DOI:** 10.1186/1471-2180-11-27

**Published:** 2011-02-02

**Authors:** Christian Gortazar, Maria J Torres, Pelayo Acevedo, Javier Aznar, Juan J Negro, Jose de la Fuente, Joaquín Vicente

**Affiliations:** 1 IREC National Wildlife Research Institute (CSIC-UCLM-JCCM), Ciudad Real, Spain; 2 Departamento de Microbiología, Universidad de Sevilla, Sevilla, Spain; 3 Biogeography, Diversity, and Conservation Research Team, Department of Animal Biology, Faculty of Sciences, University of Malaga. E-29071 Málaga, Spain; 4 Servicio de Microbiología, HH UU Virgen del Rocío, Sevilla, Spain; 5 Department of Evolutionary Ecology, Estación Biológica Doñana, CSIC, Sevilla, Spain; 6 Department of Veterinary Pathobiology, Center for Veterinary Health Sciences, Oklahoma State University, Stillwater, Oklahoma, USA

## Abstract

**Background:**

We describe the diversity of two kinds of mycobacteria isolates, environmental mycobacteria and *Mycobacterium bovis *collected from wild boar, fallow deer, red deer and cattle in Doñana National Park (DNP, Spain), analyzing their association with temporal, spatial and environmental factors.

**Results:**

High diversity of environmental mycobacteria species and *M. bovis *typing patterns (TPs) were found. When assessing the factors underlying the presence of the most common types of both environmental mycobacteria and *M. bovis *TPs in DNP, we evidenced (i) host species differences in the occurrence, (ii) spatial structuration and (iii) differences in the degree of spatial association of specific types between host species. Co-infection of a single host by two *M. bovis *TPs occurred in all three wild ungulate species. In wild boar and red deer, isolation of one group of mycobacteria occurred more frequently in individuals not infected by the other group. While only three TPs were detected in wildlife between 1998 and 2003, up to 8 different ones were found during 2006-2007. The opposite was observed in cattle. Belonging to an *M. bovis*-infected social group was a significant risk factor for mycobacterial infection in red deer and wild boar, but not for fallow deer. *M. bovis *TPs were usually found closer to water marshland than MOTT.

**Conclusions:**

The diversity of mycobacteria described herein is indicative of multiple introduction events and a complex multi-host and multi-pathogen epidemiology in DNP. Significant changes in the mycobacterial isolate community may have taken place, even in a short time period (1998 to 2007). Aspects of host social organization should be taken into account in wildlife epidemiology. Wildlife in DNP is frequently exposed to different species of non-tuberculous, environmental mycobacteria, which could interact with the immune response to pathogenic mycobacteria, although the effects are unknown. This research highlights the suitability of molecular typing for surveys at small spatial and temporal scales.

## Background

Identifying mechanisms of pathogen transmission, including potential environmental sources, is critical to control disease [1]. Molecular epidemiology integrates conventional epidemiological approaches with molecular techniques to track specific strains of pathogens in order to understand the distribution of pathogens in populations and environments [2]. This can be used to elucidate inter- and intra-specific transmission pathways and environmental risk factors, from individual to population, and from local to broader spatial scales.

The genus *Mycobacterium *comprises over 70 species and several subspecies. Over 30 of these can cause disease in livestock, wildlife and humans, occurring worldwide. Mycobacterial diseases such as bovine tuberculosis (bTB) have become a major sanitary and conservation problem even in relatively unmanaged natural areas across the world. Similar to other shared diseases, the existence of wildlife reservoirs is limiting the effectiveness of eradication schemes in livestock [3,4]. In bTB, known risk factors for wild ungulates include age, gender, density, spatial aggregation, intra and inter-specific contact, fencing and other habitat features as well as genetic factors [5-12]. However, most data derive from large scale studies [e. g. [3,13-18]], while detailed information at small spatial scales is still very scarce (in ungulates [19-24], in possums *Trichosurus vulpecula *[25,26]), and usually, fine associations with spatial and environmental factors are not addressed.

Even less information is available regarding the effects on wildlife and livestock of Mycobacteria Other Than Tuberculosis (MOTT). Environmental mycobacteria or MOTT include a large number of species that can cause serious illnesses in humans, particularly in immunocompromised patients [27]. For example, *Mycobacterium interjectum *has been identified as a causative agent of cervical lymphadenitis in children [28], and of cutaneous infections in immunosuppressed patients [29]. *M. xenopi *may cause pulmonary disease in humans [30], and *M. scrofulaceum *may cause cutaneous infections and lymphadenitis [27]. In humans, risk factors for MOTT infections include immunosuppression, contaminated water and aerosol exposure, and short or old age [27-29]. MOTT are widely distributed in the environment, particularly in wet soil, marshland, streams, rivers and estuaries, but each species shows different preferences [31].

Because of its habitat characteristics, extension and their sizeable wild and domestic animal populations, Doñana National Park (DNP) in Southern Spain has been proposed as a good natural laboratory for studying wildlife mycobacteriosis [21,32]. Molecular typing of *M. bovis *isolates for the period 1998-2003 showed that wildlife species in DNP were infected only with those *M. bovis *typing patterns (TPs) that were more prevalent in local cattle. Furthermore, the results were suggestive of micro-evolutionary events in the local *M. bovis *population [32]. In the same period, *M. bovis *infection prevalence in DNP was 33% in European wild boar (*Sus scrofa*), 21% in red deer (*Cervus elaphus*), and 26% in fallow deer (*Dama dama*) [32]. In a more recent study, we confirmed infection with *M. bovis *in 52% wild boar, 27% red deer and 18% fallow deer from DNP in 2006-2007, and evidenced that *M. bovis *prevalence decreased from North to South in wild boar and red deer, whereas no clear spatial pattern was observed for fallow deer [21].

Three wild ungulates coexist in DNP, wild boar, fallow deer and red deer, along with domestic cattle subjected to bTB eradication programs. We included the wild species as our study models as all are highly susceptible to bTB and are known to show high prevalence in the area [21]. In addition, their different ecology and behavior peculiarities [33] can play a role in the epidemiology of mycobacteria, for example, variations in sociability or gregariousness, and scavenging habits. In addition, different habitats could provide variable environmental suitability for *M. bovis *persistence [6,34]. In this sense, scrublands and woodlands are preferably used by red deer and wild boar compared with fallow deer [35-37].

In this study we used molecular epidemiological techniques to establish the extent of *M. bovis *strain richness and other environmental mycobacterial species in isolates collected in wildlife and cattle from the DNP, so as the association with social, spatial and environmental factors in this multi-host and multi-pathogen situation. We hypothesized that infection by mycobacteria would differ among hosts and sites depending on host, pathogen and local environment ecology. Fine-tuning mycobacterial epidemiology in DNP allowed rising a number of relevant questions: (1) Do hosts get infected twice by *M. bovis *and MOTT, and can this interfere in *M. bovis *infection or vice versa? (2) Have new *M. bovis *types appeared or have any changes in type composition taken place in recent years? (3) Is there an effect of the social group on infection risk? (4) Is there a spatial structure in mycobacteria distribution? (5) Are there species-specific variants of mycobacteria that could be attributed to species-specific behavior patterns (including inter-specific interaction) and/or to advanced host species-pathogen interactions?

## Methods

### Study area

The study was carried out in DNP, located in south-western Spain (37°0' N, 6°30' W) and covering 54,000 Ha. This is a flat region of sandy soils bordering the Atlantic Ocean, with a maximum elevation of 47 m. The climate is Mediterranean sub-humid with marked seasons. In the wet season (winter and spring), most of the marshlands are flooded and wildlife and cattle tend to graze in the more elevated scrublands [37]. In summer, the wetter and more productive ecotone between the scrublands and the marshes supports aggregations of wild and domestic ungulates. Human access is restricted and management is carried out by Park authorities. Limited traditional exploitation of some natural resources, such as logging, and cattle and horse rising are allowed. After 1994, when bTB in wildlife was first diagnosed in DNP a Government-sponsored program was initiated to eradicate bTB-positive cattle. Ungulate populations have been culled by shooting (between 200 and 500 individuals/year, the majority of them wild boar, or about 10-20% of the wild ungulate population estimated at 3,500 individuals).

### Animal sampling

**Figure 1:**
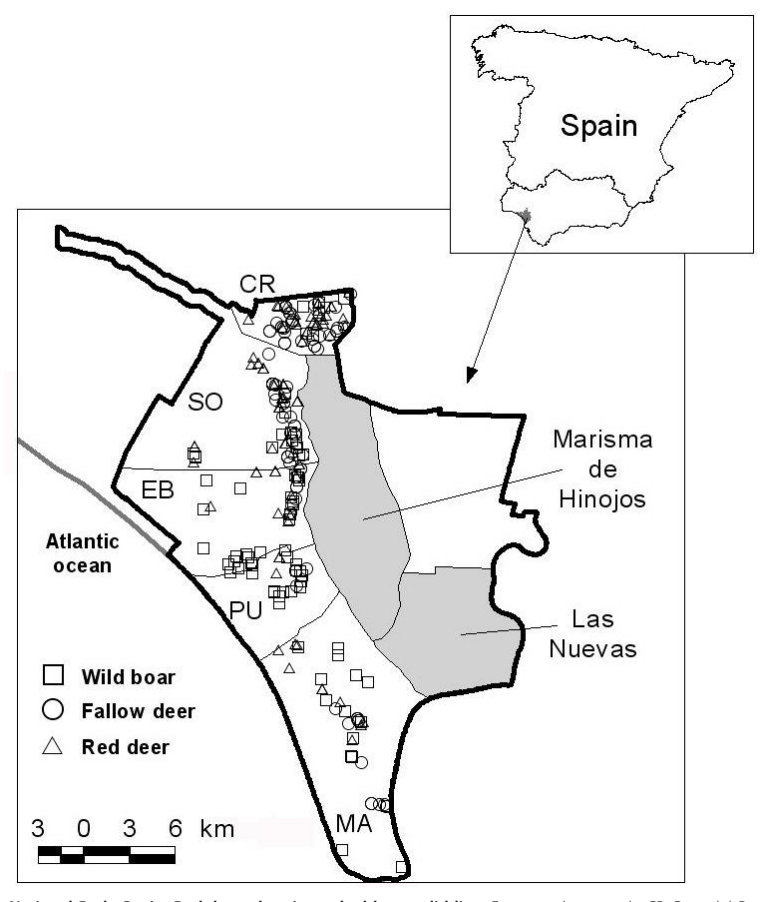
**Doñana National Park, Spain. Park boundary is marked by a solid line**. From north to south: CR Coto del Rey; SO Los Sotos; EB Estación Biológica; PU El Puntal; MA Marismillas. Shadowed areas are marshlands used as cattle pastures (Marisma de Hinojos and Las Nuevas). Symbols show sampling sites for wild boar (squares), fallow deer (circles) and red deer (triangles).

From April 2006 to April 2007, 124 European wild boar, 95 red deer, and 100 fallow deer were sampled within the park by shooting. The culling of wild ungulates was approved by the Research Commission of Doñana National Park in accordance with management rules established by the Autonomous Government of Andalucía. For each animal we recorded the exact position with GPS. Sex and age, based on tooth eruption patterns (animals less than 12 months old were classified as juveniles, those between 12 and 24 months as yearlings, and those more than 2 years old as adults; [38]), were recorded in the field. A necropsy was performed on site and the presence of tuberculosis-like lesions recorded by macroscopic inspection of lymph nodes and abdominal and thoracic organs [6]. This protocol included the examination of the lungs for the presence of TB-compatible macroscopic lesions during field inspection and a sample was collected. A tonsil and a head lymph node sample from each individual were collected for culture (Figure 1; Table 1). In wild boar, one piece of the tonsils and one from both mandibular lymph nodes were submitted for microbiological studies. In red deer and fallow deer, one piece of the tonsils and head lymphnode samples, always containing at least half left and half right medial retropharyngeal lymph node, were submitted for culture. Due to logistic and budget constraints, no thoracic or abdominal lymphoid tissues were cultured except when TB-compatible macroscopic lesions were evidenced.

**Table 1:** Mycobacterial identification and molecular typing results by species and sampling site within Doñana National Park (DNP), Spain (CR Coto del Rey; SO Los Sotos; EB Estación Biológica; PU El Puntal; MA Marismillas; see Figure 1 on molecular typing patterns and Figure 6 on regions within DNP).

			Mycobacteria Other Than Tuberculosis (MOTT)					*Mycobacterium bovis*								
**Host**	**Site**	**n**	***M. scr.***	***M. int.***	***M. xen.***	***M. int.***	**Total MOTT**	**A1**	**A3**	**B2**	**B5**	**C1**	**D4**	**E1**	**F1**	**Total *M. bovis***

Wild boar	CR	14						12							1	13
	SO	18	3				3	8						2		10
	EB	31	2	6		3	11	5		2						7
	PU	29	1			5	6	7		12						19
	MA	32						5		7	1					13

	Total	124	6	6		8	20	37		21	1			2	1	62
Red deer	CR	35						8	1				1			10
	SO	35	6			1	7	8				1				9
	EB	12				1	1	2		1						3
	PU	3						1		1						2
	MA	10				1	1									

	Total	95	6			3	9	19	1	2		1	1			24
Fallow deer	CR	36	2				2	7						1		8
	SO	35	9		1		10	8					2			10
	EB	9	3			1	4	2								2
	PU	5	2				2	1								1
	MA	15														

	Total	100	16		1	1	18	18						3		21

	**TOTAL**	**319**	**28**	**6**	**1**	**12**	**47**	**74**	**1**	**23**	**1**	**1**	**1**	**5**	**1**	**107**

M. scr. = Mycobacterium scrofulaceum; M. int. = Mycobacterium interjectum, M. xen. = Mycobacterium xenopi, M. int. = Mycobacterium intracellulare

**Table 2:** Infection with *Mycobacterium bovis*, Mycobacteria Other Than Tuberculosis (MOTT), or *M. bovis*/MOTT co-infection in wildlife hosts from Doñana National Park, Spain.

	MOTT pos		MOTT neg	
	
Host	*M. bovis *pos	*M. bovis *neg	*M. bovis *pos	*M. bovis *neg
Red deer	1	8	26	60
Fallow deer	3	15	19	63
Wild boar	4	16	57	47

Social groups were defined as animals sampled the same day at the same site, and with characteristics that were compatible with forming a stable (e.g. female-yearling) or seasonal (e.g. rut mixed) group. Only part of the individuals belonging to a given social group was sampled. Sampling was performed according to European (86/609) and Spanish laws (RD 223/1988; RD 1021/2005), and current guidelines for ethical use of animals in research (ASAB, 2006) and UCLM animal experimentation committee.

### Microbiological procedures

Culture: The specimens were liquefied and decontaminated with an equal volume of N-acetyl-L-cysteine and 2% NaOH, mixed by centrifugal swirling, and then incubated for 15 min (24). The reaction was neutralized by adding 0.0067M phosphate-buffered saline (pH 6.8), to a final volume of 50 mL. The specimens were concentrated by centrifugation at 3,000 × g for 15 min. The supernatant was discarded, and the sediment was re-suspended in 0.5 mL of sterile water. The sediment was used to inoculate two Löwestein-Jensen with pyruvate solid medium. Lowëstein-Jenssen slants were incubated at 37°C for 6 weeks and inspected weekly for growth. When growth was detected, a smear was prepared to confirm the presence of acid-fast bacilli from suspect colonies by Ziehl-Neelsen staining.

### Identification

We identified *M. bovis *and MOTT to the species level and characterized *M. bovis *strains with spoligotyping and MIRU-VNTR typing.

Macroscopic morphology of the colonies and pigment production was recorded. Identification at species level was performed with the GenoType^®^MTBC (Haim lifescience GmbH, Germany) for the *Mycobacterium *complex strains that allows the differentiation of *M. africanum *I, *M. bovis *BCG, *M. bovis *ssp. *bovis*, *M. bovis *ssp. *caprae *and *M. tuberculosis*/*M. africanum *II/*M. canettii*. MOTT strains were identified by the GenoType^® ^*Mycobacterium *CM and Genotype^® ^*Mycobacterium *AS MTBC (Haim lifescience GmbH, Germany). The GenoType assays were performed according to the manufacturer's instructions: DNA extraction by the DNA SSS method (REAL, DURVIZ, Valencia, Spain) was followed by PCR amplification of a trait of the 23S rRNA gene, as recommended. Reverse hybridization and detection were carried out on a shaking water bath (TwinCubator; Hain lifescience GmbH, Germany). The final identification was obtained by comparison of line probe patterns with the provided evaluation sheet [39].

### Typing

The *M. bovis *isolates were further characterized by spoligotyping [40]. The amplified product was detected by hybridization of the biotin-labelled PCR product onto spoligotyping membrane (Isogen Bioscience BV, Maarssen, The Netherlands). Purified sterile water and chromosomal DNA of *M. tuberculosis *H37Rv and *M. bovis *BCG P3 were included as controls in each batch of tests. The patterns were allocated a number in the *M. bovis *spoligotyping database. The results were recorded in SB (spoligotype bovis) code, followed by a field of 4 digits as defined on the *M. bovis *Spoligotype Database website (http://www.mbovis.org).

All wildlife isolates (n = 107) were also subjected to MIRU-VNTR analysis (Table 1). Extensive documentation (online, Adobe PDF manual, and Flash tutorials) on the service and the genotyping methods is available at the MIRU-VNTRplus website (http://www.miru-vntrplus.org).

**Figure 2:**
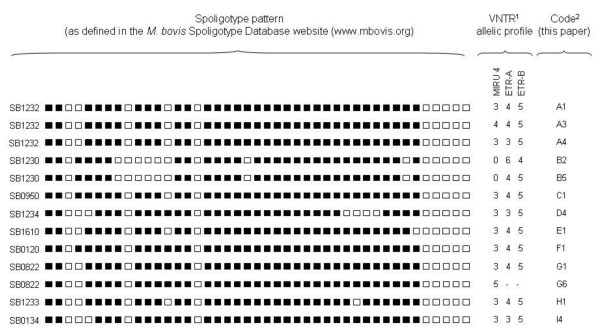
**Spoligotyping patterns, VNTR allelic variants, and codes used to define typing patterns (TPs) in this study**. 1) VNTR allelic variants for MIRU10 were always 2, for MIRU16 always 3, for MIRU23 always 4, for MIRU26 always 5, for MIRU31 always 3 and for MIRU40 always 2. 2) Isolates with TP codes A4, G1, G6, H1 and I4 as in Romero *et al*. (2008).

The analysis was carried out as described [41,42], and primers previously reported were used to amplify the ETR-A and ETR-B loci (formally VNTR 2165 and VNTR 2461) [43], MIRU 4, 10, 16, 23, 26, 31 and 40 [14] using the recommended PCR conditions. Amplicon sizes were estimated by electrophoresis on a 1.5% agarose gel at 45 V during 2 h, using 100-bp ladder (Biotools B&M). Figure 2 presents the spoligotyping patterns, VNTR allelic profiles and typing pattern (TP) codes defined for this study.

### Statistics

Chi-square tests were used for between-pair comparisons of prevalences. To test for the effect of host species *vs *site regarding the mycobacterial isolates, we used the Czechanovsky similarity index [44]. This index considers the list of mycobacterial species recorded in a given host type or in a given study area. It is calculated by dividing two times the species shared between two lists, by the total number of species of both lists, as follows:

Cz=2*(number of species shared between two lists)/(number of species of both lists)

Considering the animals in which any mycobacterial infection was diagnosed, three generalized linear mixed models (GLMM, SAS 9.0 software, GLIMMIX procedure) were explored to test different explanatory variables that affect the presence of a mycobacterial type or group. The most common mycobacterial groups were: (i) *M. bovis *(ii) *M bovis *A1 and (iii) *M. scrofulaceum*. The presence or absence of infection in a mycobacterial group was considered as a binary variable. The model was fitted using a logit link function. The model considered social group as a random effect. The model included host species (wild boar, fallow deer and red deer), the study area and age (juvenile: less than 2 years, adult: older than 2 years) as categorical explanatory variables. The distance to the water (log_10_-trasnformed) was included as a continuous predictor. To compare the spatial associations of infection by specific mycobacterial type and hosts, we included as explanatory continuous variable the ratio (log_10_-transformed) between the nearest neighbor distance from host to a different host species with the same type of mycobacteria relative to the nearest distance to a con-specific host with the same type of mycobacteria (calculated using ArcGis version 9.2, ESRI, Redlands, CA). A ratio >1 indicates that the nearest distance to a host with the same spoligotype is higher for a different host species. All the aforementioned explanatory variables we also included in the models interacting with the host species. Due to over-parameterization of the models and zero inflated data, no interactions were included in the *M. bovis *A1 and *M. scrofulaceum *models. *P*-value was set as ≤ 0.05. We estimated exact confidence limits for prevalence (proportions) using Sterne's exact method.

## Results

### Mycobacteria species and molecular types

**Figure 3:**
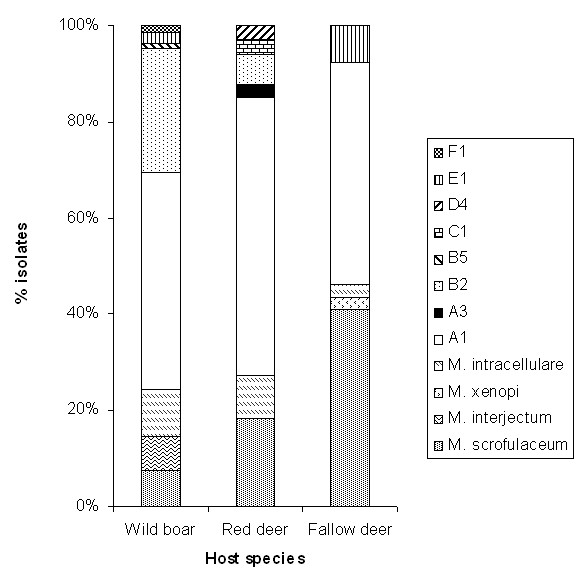
**Mycobacterial isolates (in %) identified in red deer, fallow deer and wild boar from Doñana National Park, Spain**. A1 to F1 are *Mycobacterium bovis *isolates as defined in Figure 1.

We obtained a total of 154 mycobacterial isolates from DNP wildlife. This included 107 *M. bovis *isolates belonging to 8 different typing patterns (spoligotyping pattern + VNTR profile, TP), and 47 isolates belonging to four MOTT (Table 1). *M. bovis *TPs and MOTT species were isolated from wild boar (n = 82 isolates), red deer (n = 33 isolates), and fallow deer (n = 39 isolates) (Figure 3). Wild boar and red deer had 5 *M. bovis *TPs each, whereas fallow deer presented only 2 TPs. The number of different isolates per host (MOTT and *M. bovis *TPs combined) was 8 in wild boar, 7 in red deer and 5 in fallow deer (Table 1).

Regarding *M. bovis*, we identified 6 different spoligotyping patterns and 5 different VNTR allelic profiles (Figure 2). One spoligotyping pattern was new according to the *M. bovis *database, and was therefore introduced with code SB1610. Co-infection of a single host by two *M. bovis *TPs occurred in all three wild ungulate species. One adult male red deer was infected with TPs A1 and B2, one adult male and one adult female fallow deer were co-infected with TPs A1 and E1, and two wild boar (weaner and juvenile) were co-infected with TPs A1 and B2.

MOTT species found in wildlife hosts included *M. scrofulaceum *(28 isolates) and *M. intracellulare *(12 isolates), both found in all host species, *M. interjectum *(6 isolates, only in wild boar), and *M. xenopi *(1 isolate in a fallow deer; Table 1). In four deer and four wild boar, *M. bovis *and MOTT were isolated concurrently (6 *M. scrofulaceum*, 1 *M. interjectum *and 1 *M. intracellulare*). In a single wild boar, both types of mycobacteria were simultaneously isolated from the two tissue samples collected and cultured, while in the remaining cases *M. bovis *was isolated from either lymph nodes or tonsils and the MOTT from the tissue where *M. bovis *was absent. We recorded no cases of co-infection by different MOTT.

Table 2 presents the relationship between MOTT and *M. bovis *isolation in wildlife. In cattle from DNP sampled in 2006-07, all isolates corresponded to the two dominant *M. bovis *spoligotyping patterns: spoligotype A (SB1232) in 32 cases and spoligotype B (SB1230) in 15 cases. This proportion was not significantly different from the proportion observed among wild ungulates (75 spoligotype A, 24 spoligotype B, 8 other spoligotyping patterns; Chi-square = 4.7, 2 d.f., n.s.). Only one MOTT (*M. intracellulare*) was isolated from cattle.

**Table 3:** Molecular typing patterns of *Mycobacterium bovis *isolates obtained from Doñana wildlife and cattle in 1998-2003 (drawn from Romero *et al*., 2008) and in 2006-2007 (present study).

	1998-2003					2006-2007		
	Wild boar	Red deer	Fallow deer	Other wildlife	Cattle	Wild boar	Red deer	Fallow deer
A1	14	8	13	4	22	37	19	18
A3							1	
A4					1			
B2	6	2	1	1	8	21	2	
B5						1		
C1	1			1			1	
D4					2		1	
E1						2		3
F1					1	1		
G1					1			
G6					2			
H1					1			
I4					2			

The proportion of wild ungulate *M. bovis *typing patterns (TPs) other than the dominant A1 and B2 did not differ statistically from 1998-2003 to 2006-2007 (2.2 ± 4.3% in 1998-2003, 9.3 ± 5.5% in 2006-2007, Chi-square = 2.39, 1 d.f., n.s., confidence limits are calculated according to Sterne's exact method). No spoligotyping patterns other than the two dominant ones (A and B) were detected among 47 cattle isolates in 2006 and 2007.

### Changes in mycobacterial typing patterns over time in DNP

All three *M. bovis *typing patterns recorded in DNP wildlife between 1998 and 2003 (A1, B2, C1) were still evidenced in similar proportions in 2006-2007 (Chi-square = 0.5, 2 d.f., n.s.). However, while only three different TPs had been detected in DNP wildlife in the first period, up to 8 different ones were found in the second period (Table 3). Two of these "new" TPs (D4 and F1) had already been recorded in cattle sampled in DNP between 1998 and 2003. However, 3 other TPs (A3, B5, and E1) had never before been reported in DNP.

**Table 4:** Spoligotyping patterns of *Mycobacterium bovis *isolates from Doñana cattle, by zone.

Zone	A	B
Marisma de Hinojos (Large, N to S ranging Marshland)	7	3
Los Sotos (SO)	7	2
El Puntal (PU)	5	5
Las Nuevas (Southern Marshland, close to MA and PU)	6	3
Zone not known	7	2
Total	32	15

In contrast with the situation in wildlife and to data from 1998-2003, when 10 out of 41 cattle spoligotyping patterns were different from A and B, no spoligotyping patterns other than the two dominant ones (A and B, Table 4) were detected among 47 cattle isolates in 2006 and 2007 (Chi-square = 12.9, 3 d.f., p < 0.001).

**Table 5:** Czechanovsky similarities (in %) (from north to south, CR Coto del Rey; SO Los Sotos; EB Estación Biológica; PU El Puntal; MA Marismillas) and host species (WB wild boar; RD red deer; FD fallow deer) in DNP.

	CR	SO	EB	PU	MA	WB	RD	FD
CR	-	50	36	40	20	57	62	54
SO		-	55	60	40	57	62	91
EB			-	89	67	77	67	60
PU				-	75	67	73	67
MA					-	67	54	44
WB						-	53	61
RD							-	50
FD								-

### Spatial structure

**Figure 4:**
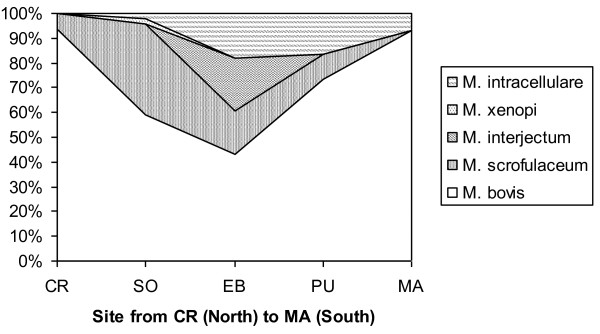
**Spatial structure of Mycobacteria Other Than Tuberculosis (MOTT) and *Mycobacterium bovis *isolates from wild ungulates in Doñana National Park, Spain**. MOTT were proportionally more frequent in the central parts of the park (SO, EB, PU; see Figure 6).

**Figure 5:**
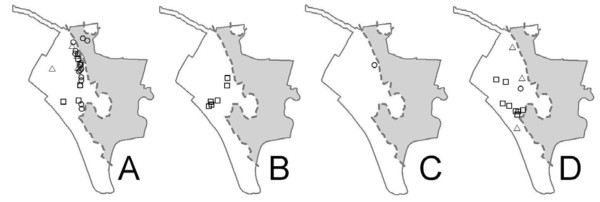
**Mycobacteria Other Than Tuberculosis (MOTT) isolates by host and site in Doñana National Park, Spain (A *Mycobacterium scrofulaceum*; B *M. interjectum*; C *M. xenopi*; D *M. intracellulare*)**. The solid line indicates the park limit and the dashed line the marshland (dark area) waterline. Symbols show sampling locations for wild boar (squares), fallow deer (circles) and red deer (triangles).

Regarding the MOTT (Table 1, Figures 4 and 5), *M. interjectum *was only found in wild boar from EB, in the central part of DNP. In contrast, *M. scrofulaceum *was found in all three wildlife hosts (but not in cattle) in CR (2 isolates), SO (18), EB (5), and PU (3). The only MOTT found in cattle (one *M. intracellulare *isolate) was isolated from a cow raised in PU. *M. intracellulare *was often isolated from wild boar in PU and EB, and also from one fallow deer in EB and two red deer in SO and MA, respectively.

**Figure 6:**
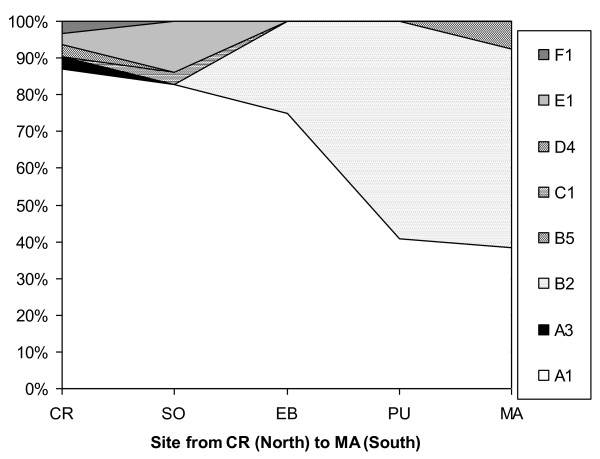
**Spatial structure of *M. bovis *isolate typing patterns (TPs) from wild ungulates in Doñana National Park, Spain. A North (CR) South (MA) gradient in type A1 and an inverse one in type B2 are evident**.

Table 5 shows the Czechanovsky similarities between the mycobacteria isolates in different sites and host species in DNP. For example, in column and row 1 from Table 5, the similarity indices of the CR mycobacterial community (in the north of DNP) decrease towards the south of the Park (MA; 20%; see also Figure 6). The highest similarity indices were observed between neighboring sites such as between EB and PU (89%) and MA and PU (75%). All hosts had their highest similarities with mycobacterial communities from the central sites of DNP.

**Table 6:** Czechanovsky similarities (in %) between the mycobacteria isolates in wild boar, red deer and fallow deer from CR (WBcr, RDcr, FDcr), wild boar, red deer and fallow deer from the remaining sites of DNP (WBr, RDr, FDr), and the remaining host species from the CR site (red and fallow deer RDFDcr; wild boar and fallow deer WBFDcr; wild boar and red deer WBRDcr)

	WBr	RDr	FDr	RDFDcr	WBFDcr	WBRDcr
WBcr	22			29		
RDcr		25			29	
FDcr			75			29

Table 6 shows the Czechanovsky similarities between the mycobacteria isolates in wild boar, red deer and fallow deer from CR (WBcr, RDcr, FDcr), wild boar, red deer and fallow deer from the remaining sites of DNP (WBr, RDr, FDr), and the remaining species from the CR site (red and fallow deer RDFDcr; wild boar and fallow deer WBFDcr; wild boar and red deer WBRDcr). The highest similarity occurred between fallow deer from CR and from the remaining parts of DNP (75%).

**Table 7:** Mycobacteria species and *Mycobacterium bovis *typing patterns (TPs) isolated from wild boar (WB), red deer (RD) and fallow deer (FD) presumptive social groups in Doñana National Park (f-fawn; y-yearling; w-weaner; ad-adult; ♀-female; ♂-male; numbers before a colon indicate more than one individual of same characteristics).

Code-Area	Group	Code-Area	Group
WB1-MA	♀-ad-A1; ♂-y-B2	RD10-EB	♀-ad-(-); ♀-ad-A1

WB2-MA	3: ♂-f-(-); ♀-f-(-); 2: ♀-ad-(-); ♀-ad-B2	RD11-SO	♀-ad-C1; ♀-ad-A1

WB3-MA	♂-y-B2; ♂-y-(-)	RD12-SO	♀-f-(-); ♀-ad-*scrofulaceum*, ♀-ad-*intracellulare*

WB4-MA	2: ♂-w-A1; ♂-w-(A1+B2)	RD13-CR	2: ♀-ad-(-); ♀-y-(-)

WB5-MA	2: ♀-ad-(-); ♀-y-(-); m-y-(-)	RD14-CR	2: ♀-ad-(-); ♀-y-*M. bovis*

WB6-PU	♀-ad-B2; ♂-w-B2	RD15-CR	♀-y-(-); ♀-ad-(-)

WB7-PU	♀-ad-(-); ♀-ad-*intracellulare*	RD16-CR	♂-ad-A1; ♂-ad-D4

WB8-PU	♀-ad-*intracellulare*; ♀-w-B2	RD17-PU	2: ♂-ad-(-)

WB9-EB	♀-y-A1; ♂-y-(A1+*intracellulare*)	RD18-EB	♂-ad-*intracellulare*; ♂-ad-(-)

WB10-CR	3: ♂-w-A1; 2: ♀-ad-(-)	RD19-EB	2: ♀-ad-(-)

WB11-EB	♂-ad-(-); ♀-ad-(-); ♀-ad-*scrofulaceum*	RD20-CR	2: ♂-ad-(-)

WB12-EB	♀-w-(-); ♀-w-*intracellulare*	RD21-MA	2: ♂-ad-(-)

WB13-EB	♂-y-(-); ♀-y-(-); ♂-ad-(-)		

WB14-SO	2: ♀-y-E1	FD1-SO	♂-f-A1; ♀-ad-A1

WB15-PU	♀-ad-B2; ♀-ad-A1	FD2-SO	7: ♀-ad-(-); ♀-ad-A1; ♂-f-(-)

WB16-PU	♂-ad-B2; ♂-y-B2; ♀-y-*intracellulare*	FD3-CR	♂-ad-(-); ♂-ad-A1

WB17-PU	♂-ad-A1; ♀-ad-*intracellulare*	FD4-CR	2: ♀-f-(-); 2: ♀-ad-(-)

WB18-PU	2: ♂-y-A1	FD5-SO	♂-y-*xenopi*; ♂-ad-(A1+*scrofulaceum*)

WB19-PU	♀-ad-(-); ♂-w-B2; ♂-w-*intracellulare*	FD6-SO	♀-y-A1; ♀-ad-(-)

WB20-CR	♂-y-A1	FD7-CR	♀-y-(-); ♀-ad-(A1+E1)

WB21-CR	♂-ad-A1	FD8-CR	♀-y-(-); ♂-y-A1

WB22-PU	2: ♀-w-B2; ♀-ad-(-)	FD9-CR	2: ♀-y-(-)

WB23-MA	♂-ad-(-); ♀-ad-A1	FD10-MA	2: ♂-ad-(-)

WB24-SO	♀-w-(-); ♀-w-A1; ♂-w-*scrofulaceum*	FD11-MA	2: ♀-ad-(-); ♂-ad-(-)

WB25-PU	♂-ad-*scrofulaceum*; ♂-ad-(-)	FD12-EB	2: ♂-ad-(-); ♂-y-A1; ♀-ad-*intracellulare*

WB26-CR	♀-ad-A1; ♂-y-A1	FD13-SO	♂-ad-(A1+E1); ♂-ad-E1

		FD14-EB	♂-y-(-); ♀-ad-(-)

RD1-SO	♂-y-(-); ♂-ad-(-)	FD15-CR	2: ♀-ad-(-); ♀-ad-A1; ♀-y-(-); ♀h-f-(-)

RD2-SO	2: ♂-ad-(-)	FD16-PU	♂-ad-(-); ♂-ad-*scrofulaceum*

RD3-CR	2: ♂-ad-A1	FD-17-SO	♂-ad-(-); ♂-ad-*scrofulaceum*

RD4-SO	♀-y-(-); ♂-ad-(-)	FD18-CR	2: ♂-ad-(-)

RD5-CR	2: ♂-ad-(-)	FD19-SO	♀-f-*scrofulaceum*; ♀-ad-A1; ♀-y-(-); ♀-ad-(-)

RD6-SO	5: ♂-ad-(-)	FD20-MA	2: ♀-ad-(-); ♀-f-(-)

RD7-SO	♀-y-(-); ♀-ad-A1; ♂-ad-A1	FD21-MA	♂-ad-*M. bovis*; ♂-ad-(-)

RD8-CR	♀-ad-(-); ♂-ad-(-)	FD22-CR	2: ♂-ad-A1

RD9-SO	♂-ad-A1; ♀-y-A1	FD23-CR	♂-y-(-); ♂-ad-(-)

TP identification (A1...E1) as in Figure 2.

### Factors affecting the presence of *M. bovis *TPs and MOTTs

Social groups, defined as animals sampled the same day at the same site, and with characteristics that were compatible with forming a stable (e.g. female-yearling) or seasonal (e.g. rut mixed) social group were characterized (Tables 7 and Table 8). In total, 52% of 69 social groups were infected with *M. bovis*. Among red deer, 21 groups were identified, *M. bovis *infecting deer in one third of these groups (n = 7; 33%). In fallow deer, we identified 22 groups with 12 (54%) including at least one *M. bovis *infected individual. Up to 26 wild boar groups were identified, and 20 (77%) were found infected with *M. bovis*. Regarding the proportion of social groups with more than one TP, no between host species statistical differences were evidenced. After eliminating one infected individual per group, red deer (Chi-square = 16.1, 1 d.f., *p*<0.001) and wild boar Chi-square = 7.7, 1 d.f., *p*<0.01) from infected groups were more probably infected than con-specifics from non infected ones but this did not occur in fallow deer (Chi-square = 1.4, 1 d.f., n.s.).

**Table 8:** *Mycobacterium bovis *isolates and typing patterns (TPs) from presumptive social groups of red deer, fallow deer and wild boar from Doñana National Park, Spain.

Host	n	% groups infected	Not infected	Single isolate	Multiple isolates	
					**One TP**	**Several TPs**

Red deer	21	33.3	14	2	3	2
Fallow deer	22	54.5	10	9	2	2
Wild boar	26	76.9	6	7	10	3
Total	69	52.1	30	19	15	6

**Table 9:** Estimates of parameters and standard errors for different variables that affect the presence of (i) *Mycobacterium bovis *(ii) *M. bovis *A1 and (iii) *M. scrofulaceum*.

(i) *M. bovis*			
	**d.f. num/den**	**Parameter estimates ± S.E**.	***P*-value**

**Host species**	2/1	RD = 0.7 ± 14.6, FD = -15.0 ± 17.4	0.99

**Area**	4/1	CR = 8.2 ± 37.9, EB = 0.4 ± 2.3, MA = -10.4 ± 28.8, PU = 0.99 ± 2.0	0.96

**Age**	1/85	0.8 ± 0.7	0.24

**Distance to marsh**	1/78	2.7 ± 2.9	0.03

**Distance to other host species similarly infected**	1/94	-1.3 ± 0.4	0.19

Host species*area	2/74	Not shown	0.53

Host species*Distance to marsh	7/1	RD*distance = 0.5 ± 4.5, FD*distance = 6.3 ± 5.7	0.96

Distance to other host sim. inf. *host species	2/95	RD*distance = 2.2 ± 1.2, FD*distance = 3.8 ± 1.1	0.002

**(ii) *M. bovis *A1**			

**Host species**	2/103	RD = -0.8 ± 1.2, FD = -2.1 ± 1.1	0.18

**Area**	4/97	EB = -0.9 ± 1.2, MA = -3.0 ± 1.5, PU = -2.8 ± 1.2	0.008

**Distance to marsh**	1/97	-1.7 ± 1.3	0.20

**Distance to other host species similarly infected**	1/111	0.1 ± 0.2	0.81

**(iii) *M. scrofulaceum***			

**Host species**	2/87	RD = 2.4 ± 1.8, FD = 6.3 ± 1.7	0.001

**Area**	4/85	CR = -5.4 ± 1.9, EB = -1.2 ± 1.7, MA = -9.8 ± 13.0, PU = -2.0 ± 2.3	0.08

**Distance to marsh**	1/72	2.1 ± 1.9	0.26

**Distance to other host species similarly infected**	1/119	0.8 ± 0.4	0.03

Reference levels for 'Area' and 'Host species' are 'SO (Sotos)' and 'wild boar' respectively.

FD = fallow deer, RD = red deer. CR = Coto del Rey, EB = Estación Biológica, MA = Marismillas, PU = El puntal.

Statistics concerning the GLMMs to test the factors affecting the presence of a given mycobacterial type or group are shown in Table 9. Concerning the *M. bovis vs *MOTT GLMM, the distance to water was statistically higher in MOTT infected individuals than in *M. bovis *ones (MOTT mean distance to water = 1989 ± 245 m; *M. bovis *mean distance to water ± SD = 1513 ± 164 m). The ratio of the minimum distances to similarly infected hosts (which in average were always higher than 1 for the three host species and analyzed mycobacterial groups) statistically interacted with the host. The ratios (log_10_-trasnformed) were similar for MOTT and *M. bovis *in both deer species (2.13 ± 0.36 and 2.11 ± 0.32 for MOTT and *M. bovis *in red deer; 2.01 ± 0.11 and 1.95 ± 0.35 m for MOTT and *M. bovis *in fallow deer), whereas they were higher for *M. bovis *than MOTT in the wild boar (2.71 ± 0.36 and 3.55 ± 0.20 m for MOTT and *M. bovis*). This would indicate that in wild boar the intraspecific spatial aggregation of *M. bovis *is higher than for MOTT.

When attending to specific mycobacterial types, there were statistical differences between zones for *bovis *TP A1, so that it was dominant in wild ungulates from the north of DNP (Table 1, Figure 6). There were statistical differences in the probability of infection by *M. scrofulaceum *relative to other types among host species (wild boar = 7.3%; Red deer = 18.2% and fallow deer = 41.0%; Table 1). *M. scrofulaceum *presented a lower intraspecific spatial aggregation than the remaining mycobacterial types (2.19 ± 0.20 and 2.90 ± 0.15 m ratios for *M. scrofulaceum *and the remaining types, respectively).

## Discussion

This study provided new insights into the ecology of *M. bovis *and environmental mycobacteria in complex host and pathogen communities, showing that mycobacteria are structured by host species and sampling site, even at very small spatial scales. The study also showed that host species differences in spatial patterns may greatly depend on behavioral and/or specific host-pathogen-environment interactions, for which our molecular and ecological approach allowed obtaining valuable information on the involved risk factors.

### Mycobacterial species and typing patterns

Contrary to most previous studies in wildlife, where single TPs tend to dominate in each geographical region [e.g. [19,20,45]] we detected a high richness of both MOTT and *M. bovis *TPs in DNP. Whereas single TPs are indicative of single introduction events of *M. bovis*, in our case the high identified TP richness is probably a consequence of (i) historical cattle breeding and consequent exchanges with breeders from outside the park, (ii) variable conditions provided by high environmental diversity, and (iii) the diversity and abundance of suitable wildlife hosts.

Multiple infection of a wildlife host with several *M. bovis *TPs had recently been found in one wild boar from this study area [32]. This observation is rare in wildlife *M. bovis *hosts [46]. To the best of our knowledge, this is the first study reporting co-infection of red deer and fallow deer with several *M. bovis *TPs. Moreover, the efficiency of isolating mycobacteria could have been improved with the inclusion of liquid media, suggesting that we detected only part of the true co-infections. The relevance of these findings is that they demonstrate that *M. bovis *infected wildlife hosts may become infected more than once under natural conditions, at least in areas of high infection pressure such as DNP. These results also suggest that cross-protection between different *M. bovis *strains is limited, further underlining the importance of genetic factors rather than immune responses in controlling mycobacterial infections in wildlife [11,47,48]. Additionally, the infection exclusion reported for closely related genotypes of other intracellular bacteria of the genus *Anaplasma *[49] did not appear to occur for *M. bovis *TPs.

Co-existence of members of the *M. tuberculosis *complex and MOTT, such as *M. intracellulare*, had already been reported in human patients [50]. As previously discussed, the fact that we found several *M. bovis *- MOTT co-infections suggests that infection by one organism does not impede infection by the other in these wildlife host species. However, in all three wildlife hosts, isolation of one group of mycobacteria occurred more frequently in individuals not infected by the other group, suggesting that either some competition between mycobacteria or some laboratory bias towards the first identifiable growth may exist. This is also suggested by the finding that in all cases but in one, *M. bovis *was isolated from either lymph nodes or tonsils and the MOTT from the tissue where *M. bovis *was absent. In humans, it has been suggested that BCG vaccination protects children against cervical lymph node infection by MOTT [27].

Several authors have reported infection of wild boar with *M. scrofulaceum*, *M. interjectum*, *M. xenopi *and *M. intracellulare *[51,52]. All four MOTT recorded in this study had also already been reported in other wildlife species [18,53]. However, this is the first report of *M. xenopi *in deer.

### Changes over time in DNP

Apparently, the community of *M. bovis *in domestic cattle lost strain richness from time one (1998-2003) to time two (2006-2007), which may result from the application of the official test and slaughter program. However, the alternative hypothesis of some rare strains going undetected at any sampling period cannot be completely excluded. Part of the new TPs isolated from wildlife had been reported in cattle in the earlier survey (D4, F1). This suggests cases of spill-over from cattle to wild ungulates, and subsequent maintenance of these TPs in wildlife reservoir hosts. Other TPs had been detected neither in DNP cattle nor in wildlife, but are widespread in Spain (e.g. F1, SB0120). This would suggest a recent introduction, possibly via infected cattle. However, TP E1 is of particular interest. This TP had never been detected, but is similar to the dominant TP A1 except for one spacer. More sampling and long term studies are needed in order to test whether pathogen evolution resulted in higher TP richness in wildlife species when compared to cattle [32].

### Spatial structure

Our finding that different wildlife species were infected with the same types at a very local scale suggests that transmission is likely to occur between the species. Fallow deer differed from red deer and wild boar in showing more homogeneity in their mycobacterial isolates, regardless of the sampling area. This may be due to a higher rate of movement of fallow deer between areas and therefore relates to specific territorial and aggregation behaviors as commented above. This in turn would be relevant for disease control, suggesting a higher capacity of this host for spreading pathogens over long distances. The different distribution patterns of *M. bovis *TPs may be due to historical introduction of different TPs, presumably by infected cattle, in different parts of DNP or, alternatively, if environmental survival of mycobacteria plays a role, to a better adaptation of certain TPs to the varying habitat characteristics of northern and southern DNP.

### Factors affecting the presence of *M. bovis *TPs and MOTTs

In a previous paper we found that infection risk in wild boar was dependent on wild boar *M. bovis *prevalence in the buffer area containing interacting individuals. However, this was not evidenced for deer [21]. In this study, using the identification of social groups as an alternative tool, we found that red deer and wild boar were more prone to be infected with *M. bovis *if they were part of a group with at least one infected deer, while as commented above, this was not the case for fallow deer. High intraspecific transmission rates at early ages within wild boar social groups have been suggested in wild boar from Spain [6], and this probably relates to close interaction when foraging or routing. Animal behavior is an important aspect of disease/host dynamics that as yet has not been well documented but may play an important role in the transmission in free-ranging wildlife populations [33]. Owing to higher contact rates and common environmental risk factors, bTB transmission should occur more frequently within certain social groups. Recently, [1] used host population genetics to show that contact within family groups probably was a significant mechanism of *M. bovis *transmission among white-tailed deer (*Odocoileus virginianus*) in Michigan (USA). In DNP, modelling suggested that wild boar infection probability depends on wild boar bTB prevalence in a buffer zone of interacting individuals, while no such effect was observed in deer [21].

The fallow deer was the only species whose mycobacterial community showed more intra-species similarity throughout DNP than site similarity. Although fallow deer displayed the lowest prevalence (which is probably related to a lower natural host susceptibility, [21]), its highly gregarious behavior and subsequent increased transmission risk (at least during seasonal rutting) may cause mycobacterial strains to be shared by many social groups after social disruption. This is consistent with the finding that fallow deer displayed the lowest *M. bovis *prevalence, but a disproportionally high social group prevalence (i.e. spread across population subunits) as compared to that of red deer. That is, the findings that fallow deer belonging to groups with infected individuals were only rarely infected, or that most infected fallow deer groups had only one infected animal, strongly suggest that either the intra-specific intra-group transmission rate or the susceptibility of fallow deer to bTB is lower than in red deer. However, the alternative explanation that culturing from head lymphoid tissue only missed to detect infection disproportionally more in fallow deer than in red deer or wild boar cannot be excluded.

Confirming the above discussed, a spatial structuring in the mycobacterial isolates was evidenced for *M. bovis *A1 type, so that it was dominant in wild ungulates from the north of DNP while B2 was dominant in the south (Table 1, Figure 6). When we assessed the spatial associations (measured as nearest distances to similar and other host species) of *M. bovis *TPs, MOTT, and *M. scrofulaceum*, our findings were consistent with spatial aggregation of the host species with the same types.

The spatial distribution of *M. bovis versus *MOTT was probably linked with the water, since wildlife hosts infected with *M. bovis *were most often sampled closer to the marshland than MOTT. Environmental water sources could act not only as environmental sources of mycobacteria but also by favoring closer contact between the species [7], and this could promote more the transmission of *M. bovis *by close contact than indirect transmission of MOTT, which one would expect to be more dependent on external factors.

There were statistical differences in the probability of infection by *M. scrofulaceum *relative to other types among host species. *M scrofulaceum *is a slow-growing atypical mycobacteria that is found in environmental water sources. Nonetheless, no association was evidenced with distance to marshland. We speculate that the rooting behavior of wild boar may relate to increased exposure to this mycobacteria than other hosts. Nonetheless, our study does not discard that advanced host species-pathogen interactions may also result in different relative occurrences of mycobacterial types across the studied host species.

## Conclusions

The diversity of mycobacteria described herein is indicative of multiple introduction events and a complex multi-host and multi-pathogen epidemiology in DNP. Fine-tuning the epidemiology of mycobacterial infections allowed us to answer a number of relevant questions: First, co-infection of a single host by two *M. bovis *TPs occurred in all three wild ungulate species, confirming that one host can get infected twice. Second, significant changes in the mycobacterial isolate community may have taken place, even in a short time period (1998 to 2007). Third, we confirmed that red deer and wild boar, but not fallow deer from infected social groups were more probably infected than those from non infected groups. Hence, we agree with the views of several authors suggesting that aspects of host social organization should be taken into account in wildlife epidemiology [1,8]. Fourth, we got insights of spatial structure in mycobacteria distribution, and discussed both habitat-related and host-related explanations for the observed differences. Finally, we conclude that wildlife in DNP is frequently exposed to different species of non-tuberculous, environmental mycobacteria, which could interact with the immune response to pathogenic mycobacteria, although the effects are unknown [54].

In the present study we found evidence of mixed infection, i.e., co-infection of a single host by two *M. bovis *TPs in all three wild ungulate species, and also four deer and four wild boar concurrently presented *M. bovis *and MOTT. The possibility of cross contamination at laboratory or DNA level was ruled out. Nonetheless the sensitivity of bacterial culture and DNA fingerprinting for the identification of more than one mycobacteria species or *M. tuberculosis *complex strain may be limited when the strains are not present in the particular cultured organ/tissue. In the context of the endemicity of tuberculosis and the possibility of repeated exposure to newer infections, one can expect more cases with multiple strain infection [55]. Therefore more research concerning whether infection with one strain would protect against infection with another strain is needed.

Molecular typing did not allow inferring the direction of transmission [32]. However, findings of rare TPs such as E1 among both fallow deer and wild boar strongly suggest that interspecies transmission and/or common sources of infection do occur among wild ungulates. Conversely, the lack of isolation of rare *M. bovis *spoligotype patterns from cattle of the 2006-2007 sample suggests that spill-back from the wildlife reservoir to livestock may not be a very usual event. The results highlight the suitability of molecular typing for surveys at small spatial and temporal scales. However, increased surveillance along with a better understanding of the transmission routes, environmental persistence, and associated risk factors (e.g. scavenging) are needed if we are to effectively control bovine TB in DNP. One remaining question relates to the influence of the genotype of mycobacteria on the virulence [56], which may be mediated by secondary infections, which should be addressed by future research.

## Competing interests

The authors declare that they have no competing interests.

## Authors' contributions

Conceived and designed the study: CG, MT, JN, JF, JV. Participated in sampling and field work: CG, MT, JN, JV. Carried out the laboratory work: MT, JA Analyzed the data: CG, JN, PA, JV. Draft the manuscript: CG, MT, JN, PA, JF, JV. All authors read and approved the final manuscript.

## References

[B1] Blanchong JA Scribner KT Kravchenko AN Winterstein SR TB-infected deer are more closely related than non-infected deer Biol Lett 2007 3 103 105 10.1098/rsbl.2006.0547 17443977 PMC2373800

[B2] Skuce RA Neill SD Molecular epidemiology of *Mycobacterium bovis*: exploiting molecular data Tuberculosis 2001 81 169 175 10.1054/tube.2000.0270 11463239

[B3] Aranaz A de Juan L Montero N Sanchez C Galka M Delso C Álvarez J Romero B Bezos J Vela AI Briones V Mateos A Domínguez L Bovine tuberculosis (*Mycobacterium bovis*) in wildlife in Spain J Clin Microbiol 2004 42 2602 2608 10.1128/JCM.42.6.2602-2608.2004 15184440 PMC427808

[B4] Gortázar C Ferroglio E Hofle U Frolich K Vicente J Diseases shared between wildlife and livestock: a European perspective Eur J Wildl Res 2007 53 241 256

[B5] Acevedo-Whitehouse K Vicente J Gortázar C Höfle U Fernandez-de-Mera IG Amos W Genetic resistance to bovine tuberculosis in the Iberian wild boar Mol Ecol 2005 14 3209 3217 10.1111/j.1365-294X.2005.02656.x 16101786

[B6] Vicente J Höfle U Garrido JM Fernández-de-Mera IG Juste R Barral M Gortázar C Wild boar and red deer display high prevalences of tuberculosis-like lesions in Spain Vet Res 2006 37 107 119 10.1051/vetres:2005044 16336928

[B7] Vicente J Höfle U Garrido JM Fernandez-De-Mera IG Acevedo P Juste RA Barral M Gortázar C Risk factors associated with prevalence of tuberculosis-like lesions in wild boar and red deer in South Central Spain Vet Res 2007 38 451 464 10.1051/vetres:2007002 17425933

[B8] Vicente J Höfle U Fernández-de-Mera IG Gortázar C The importance of parasite life-history and host density in predicting the impact of infections in red deer Oecologia 2007 152 655 664 10.1007/s00442-007-0690-6 17401583

[B9] Acevedo P Vicente J Ruiz-Fons JF Cassinello J Gortázar C Estimation of European wild boar relative abundance and aggregation: a novel method in epidemiological risk assessment Epid Infect 2007 135 519 527 10.1017/S0950268806007059 PMC2870594 16893488

[B10] Martin-Hernando MP Höfle U Vicente J Ruiz-Fons F Vidal D Barral M Garrido JM de la Fuente J Gortázar C Lesions associated with *Mycobacterium tuberculosis *Complex infection in the European wild boar Tuberculosis 2007 87 360 367 10.1016/j.tube.2007.02.003 17395539

[B11] Naranjo V Acevedo-Whitehouse A Vicente J Gortázar C de la Fuente J Influence of methylmalonyl-CoA mutase alleles on resistance to bovine tuberculosis in the European wild boar (*Sus scrofa*) Anim Genet 2008 39 316 320 10.1111/j.1365-2052.2008.01725.x 18454807

[B12] Naranjo V Gortazar C Vicente J de la Fuente J Evidence of the role of European wild boar as a reservoir of *Mycobacterium tuberculosis *complex Vet Microbiol 2008 127 1 9 10.1016/j.vetmic.2007.10.002 18023299

[B13] Collins DM De Lisle GW Gabric DM Geographic distribution of restriction types of *Mycobacterium bovis *isolates from brush-tailed possums (*Trichosurus vulpecula*) in New Zealand J Hyg (Lond) 1986 96 431 438 10.1017/S0022172400066201 3016075 PMC2129707

[B14] Gortázar C Vicente J Samper S Garrido J Fernandez-De-Mera IG Gavín P Juste RA Martín C Acevedo P de la Puente M Hofle U Molecular characterization of *Mycobacterium tuberculosis *complex isolates from wild ungulates in South-Central Spain Vet Res 2005 36 43 52 15610722 10.1051/vetres:2004051

[B15] Lutze-Wallace C Turcotte C Sabourin M Berlie-Surujballi G Barbeau Y Watchorn D Bell J Spoligotyping of *Mycobacterium bovis *isolates found in Manitoba Can J Vet Res 2005 69 143 145 15971679 PMC1142182

[B16] Baker MG Lopez LD Cannon MC De Lisle W Collins DM Continuing *Mycobacterium bovis *transmission from animals to humans in New Zealand Epid Infect 2006 134 1068 1073 10.1017/S0950268806005930 PMC2870481 16569268

[B17] Delahay RJ Smith GC Barlow AM Walker N Harris A Clifton-Hadley RS Cheeseman CL Bovine tuberculosis infection in wild mammals in the south-west region of England: a survey of prevalence and a semi-quantitative assessment of the relative risk to cattle Vet J 2007 173 287 301 10.1016/j.tvjl.2005.11.011 16434219

[B18] De Lisle GW Kawakami RP Yates GF Collins DM Isolation of *Mycobacterium bovis *and other mycobacterial species from ferrets and stoats Vet Microbiol 2008 132 402 407 10.1016/j.vetmic.2008.05.022 18632227

[B19] De Vos V Raath JP Bengis RG Kriek NJP Huchzermeyer H Keet DF Michel A The epidemiology of tuberculosis in free ranging African buffalo (*Syncerus caffer*) in the Kruger National Park, South Africa Onderstepoort J Vet Res 2001 68 119 130 11585089

[B20] Michel AL Coetzee ML Keet DF Maré L Warren R Cooper D Bengis RG Kremer K van Helden P Molecular epidemiology of *Mycobacterium bovis *isolates from free-ranging wildlife in South African game reserves Vet Microbiol 2009 133 335 343 10.1016/j.vetmic.2008.07.023 18786785

[B21] Gortázar C Torres MJ Vicente J Acevedo P Reglero M de la Fuente J Negro JJ Aznar J Bovine tuberculosis in Doñana biosphere reserve: the role of wild ungulates as disease reservoirs in the last Iberian lynx strongholds PLoS ONE 2008 3 e2776 18648665 10.1371/journal.pone.0002776 PMC2464716

[B22] Zanella G Durand B Hars J Moutou F Garin-Bastuji B Duvauchelle A Femé M Karoui C Boschiroli ML *Mycobacterium bovis *in wildlife in France J Wildlife Dis 2008 44 99 108 10.7589/0090-3558-44.1.99 18263825

[B23] Woodroffe R Donnelly CA Johnston WT Bourne FJ Cheeseman CL Clifton-Hadley RS Cox DR Gettinby RG le Fevre AM McInerney JP Morrison WI Spatial association of *Mycobacterium bovis *infection in cattle and badgers *Meles meles* J Appl Ecol 2005 42 852 862 10.1111/j.1365-2664.2005.01081.x

[B24] Jenkins HE Woodroffe R Donnelly CA Cox DR Johnston WT Bourne FJ Cheeseman CL Clifton-Hadley RS Gettinby G Gilks P Hewinson RG McInerney JP Morrison WI Effects of culling on spatial associations of *Mycobacterium bovis *infections in badgers and cattle J Appl Ecol 2007 44 897 908 10.1111/j.1365-2664.2007.01372.x

[B25] Collins DM DNA typing of *Mycobacterium bovis *strains from the Castlepoint area of the Wairarapa N Z Vet J 1999 47 207 209 16032105 10.1080/00480169.1999.36145

[B26] Corner LAL Stevenson MA Collins DM Morris RS The re-emergence of *Mycobacterium bovis *infection in brushtail possums (*Trichosurus vulpecula*) after localised possum eradication N Z Vet J 2003 51 73 80 16032303 10.1080/00480169.2003.36343

[B27] Primm TP Lucero CA Falkinham JO III Health Impacts of Environmental Mycobacteria Clin Microbiol Rev 2004 17 98 106 10.1128/CMR.17.1.98-106.2004 14726457 PMC321467

[B28] De Baere T Moerman M Rigouts L Dhooge C Vermeersch H Verschraegen G Vaneechoutte M *Mycobacterium interjectum *as causative agent of cervical lymphadenitis J Clin Microbiol 2001 39 725 727 10.1128/JCM.39.2.725-727.2001 11158135 PMC87804

[B29] Fukuoka M Matsumura Y Kore-eda S Iinuma Y Miyachi Y Cutaneous infection due to *Mycobacterium interjectum *in an immunosuppressed patient with microscopic polyangiitis Br J Dermatol 2008 159 1382 1384 10.1111/j.1365-2133.2008.08867.x 18808411

[B30] van Ingen J Boeree MJ de Lange WC Hoefsloot W Bendien SA Magis-Escurra C Dekhuijzen R van Soolingen D *Mycobacterium xenopi *clinical relevance and determinants, the Netherlands Emerg Infect Dis 2008 14 385 389 10.3201/eid1403.061393 18325251 PMC2570832

[B31] Grange JM Greenwood D, Slack R, Peitherer J, Barer M Environmental mycobacteria Medical Microbiology 2007 17 Elsevier 221 227

[B32] Romero B Aranaz A Sandoval A Alvarez J de Juan L Bezos J Sanchez C Galka M Fernandez P Mateos A Dominguez L Persistence and molecular evolution of *Mycobacterium bovis *population from cattle and wildlife in Doñana National Park revealed by genotype variation Vet Microbiol 2008 132 87 95 10.1016/j.vetmic.2008.04.032 18539410

[B33] Conner MM Ebinger MR Blanchong JA Cross PC Infectious Disease in Cervids of North America. Data, Models, and Management Challenges Ann NY Acad Sci 2008 1134 146 172 10.1196/annals.1439.005 18566093

[B34] Miller R Kaneene JB Fitzgerald SD Schmitt SM Evaluation of the influence of supplemental feeding of white-tailed deer (*Odocoileus virginianus*) on the prevalence of bovine tuberculosis in the Michigan wild deer population J Wildl Dis 2003 39 84 95 12685071 10.7589/0090-3558-39.1.84

[B35] Rogers PM Myers K Animal distributions, landscape classification and wildlife management, Coto Doñana, Spain J Appl Ecol 1980 17 545 565 10.2307/2402636

[B36] Braza F Álvarez F Geldof R Byloo H Desplazamientos de ungulados silvestres a través de una zona de ecotono en Doñana. Doñana Acta Vert 1984 11 275 287

[B37] Braza F Alvarez F Habitat use by red deer and fallow deer in Doñana National Park Misc Zool 1987 11 363 367

[B38] Saenz De Buruaga M Lucio AJ Purroy J Diputación Foral de Álava Reconocimiento de sexo y edad en especies cinegéticas 1991

[B39] Russo C Tortoli E Menichella D Evaluation of the New GenoType *Mycobacterium *Assay for Identification of Mycobacterial Species J Clin Microbiol 2006 44 334 339 10.1128/JCM.44.2.334-339.2006 16455880 PMC1392669

[B40] Kamerbeek J Schouls L Kolk A vanAgterveld M vanSoolingen D Kuijper S Bunschoten A Molhuizen H Shaw R Goyal M van Embden J Simultaneous detection and strain differentiation of Mycobacterium tuberculosis for diagnosis and epidemiology J Clin Microbiol 1997 35 907 914 9157152 10.1128/jcm.35.4.907-914.1997 PMC229700

[B41] Roring S Scott A Brittain D Walker I Hewinson G Neill S Skuce R Development of variable-number tandem repeat typing of *Mycobacterium bovis*: Comparison of results with those obtained by using existing exact tandem repeats and spoligotyping J Clin Microbiol 2002 40 2126 2133 10.1128/JCM.40.6.2126-2133.2002 12037076 PMC130792

[B42] Supply P Allix C Lesjean S Cardoso-Oelemann M Rüsch-Gerdes S Willery E Savine E de Haas P van Deutekom H Roring S Bifani P Kurepina N Kreiswirth B Sola C Rastogi N Vatin V Gutierrez MC Fauville M Niemann S Skuce R Kremer K Locht C van Soolingen D Proposal for standardization of optimized mycobacterial interspersed repetitive unit-variable number tandem repeat typing of *Mycobacterium tuberculosis* J Clin Microbiol 2006 44 4998 4510 10.1128/JCM.01392-06 PMC1698431 17005759

[B43] Frothingham R Meeker-O'Connell WA Genetic diversity in the *Mycobacterium tuberculosis *complex based on variable numbers of tandem DNA repeats Microbiology 1998 144 1189 1196 10.1099/00221287-144-5-1189 9611793

[B44] Margalef R Ecologia 1977 Omega

[B45] Smith NH Dale J Inwald J Palmer S Gordon SV Hewinson RG Smith JH The population structure of *Mycobacterium bovis *in Great Britain: clonal expansion Proc Nat Acad Sci USA 2003 100 15271 15275 10.1073/pnas.2036554100 14657373 PMC299979

[B46] VerCauteren KC Atwood TC DeLiberto TJ Smith HJ Stevenson JS Thomsen BV Gidlewski T Payeur J Sentinel-based Surveillance of Coyotes to Detect Bovine Tuberculosis, Michigan Emerg Infect Dis 2008 14 1862 1869 10.3201/eid1412.071181 19046508 PMC2634611

[B47] Naranjo V Ayoubi P Vicente J Ruiz-Fons F Gortázar C Kocan KM de la Fuente J Characterization of selected genes upregulated in non-tuberculous European wild boar as possible correlates of resistance to *Mycobacterium bovis *infection Vet Microbiol 2006 116 224 231 10.1016/j.vetmic.2006.03.013 16672181

[B48] Naranjo V Gortázar C Villar M de la Fuente J Comparative genomics and proteomics to study tissue-specific response and function in natural *Mycobacterium bovis *infections Anim Health Res Rev 2007 8 81 88 10.1017/S1466252307001260 17692145

[B49] de la Fuente J García-García JC Blouin EF Saliki JT Kocan KM Infection of tick cells and bovine erythrocytes with one genotype of the intracellular ehrlichia *Anaplasma marginale *excludes infection with other genotypes Clin Diagn Lab Immun 2002 9 658 668 10.1128/CDLI.9.3.658-668.2002 PMC119989 11986275

[B50] Takeda M Ito W Kobayashi N Konno K Takahashi T Tatsuko R Tomita N Tanigai T Chiba T Yamaguchi K Sato K Ueki S Kayaba H Chihara J Co-existence of *Mycobacterium tuberculosis *and *Mycobacterium intracellulare *in one sputum sample Intern Med 2008 47 1057 60 10.2169/internalmedicine.47.0511 18520121

[B51] Machackova M Matlova L Lamka J Smolik J Melicharek I Hanzlikova M Docekal J Cvetnic Z Nagy G Lipiec M Ocepek M Pavlik I Wild boar (*Sus scrofa*) as a possible vector of mycobacterial infections: review of literature and critical analysis of data from Central Europe between 1983 and 2001 Vet Med 2003 48 51 65

[B52] Zanetti S Bua A Molicotti P Delogu G Mura A Ortu S Sechi LA Identification of mycobacterial infections in wild boars in Northern Sardinia, Italy Acta Vet Hung 2008 56 145 52 10.1556/AVet.56.2008.2.1 18669241

[B53] Bercovier H Vincent V Mycobacterial infections in domestic and wild animals due to *Mycobacterium marinum, M. fortuitum, M. chelonae, M. porcinum, M. farcinogenes, M. smegmatis, M. scrofulaceum, M. xenopi, M. kansasii, M. simiae and M. genavense* Rev Sci Tech 2001 20 265 290 11288516 10.20506/rst.20.1.1269

[B54] Michel AL Hlokwe TM Coetzee ML Maré L Connoway L Rutten VPMG Kremer K High *Mycobacterium bovis *genetic diversity in a low prevalence setting Vet Microbiol 2008 126 151 159 10.1016/j.vetmic.2007.07.015 17720336

[B55] Richardson M Carroll NM Engelke E Gian D van der Spuy Salker F Munch Z Gie RP Warren RM Beyers N van Helden PD Multiple *Mycobacterium tuberculosis *strains in early cultures from patients in a high-incidence community setting J Clin Microbiol 2002 40 2750 2754 10.1128/JCM.40.8.2750-2754.2002 12149324 PMC120639

[B56] Petrelli D Sharma MK Wolfe J Al-Azem A Hershfield E Kabani A Strain-related virulence of the dominant Mycobacterium tuberculosis strain in the Canadian province of Manitoba Tuberculosis 2004 84 317 326 10.1016/j.tube.2004.01.001 15207807

